# Cocaine self-administration in mice with forebrain knock-down of *trpc5* ion channels

**DOI:** 10.12688/f1000research.2-53.v1

**Published:** 2013-02-15

**Authors:** Matthew B Pomrenze, Michael V Baratta, Kristin C Rasmus, Brian A Cadle, Shinya Nakamura, Lutz Birnbaumer, Donald C Cooper

**Affiliations:** 1 Institute for Behavioral Genetics, University of Colorado, Boulder, CO, 80309, USA; 2 National Institute of Environmental Health Science, National Institute of Health, Research Triangle Park, NC, 27709, USA

## Abstract

Canonical transient receptor potential (TRPC) channels are a family of non-selective cation channels that play a crucial role in modulating neuronal excitability due to their involvement in intracellular Ca2+ regulation and dendritic growth. TRPC5 channels a) are one of the two most prevalent TRPC channels in the adult rodent brain; b) are densely expressed in deep layer pyramidal neurons of the prefrontal cortex (PFC); and c) modulate neuronal persistent activity necessary for working memory and attention. In order to evaluate the causal role of TRPC5 in motivation/reward-related behaviors, conditional forebrain TRPC5 knock-down (trpc5-KD) mice were generated and trained to nose-poke for intravenous cocaine. Here we present a data set containing the first 6 days of saline or cocaine self-administration in wild type (WT) and trpc5-KD mice. In addition, we also present a data set showing the dose-response to cocaine after both groups had achieved similar levels of cocaine self-administration. Compared to WT mice, trpc5-KD mice exhibited an apparent increase in self-administration on the first day of cocaine testing without prior operant training. There were no apparent differences between WT and trpc5-KD mice for saline responding on the first day of training. Both groups showed similar dose-response sensitivity to cocaine after several days of achieving similar levels of cocaine intake.

## Introduction

The prefrontal cortex (PFC) supports higher order cognitive functions, such as decision-making, reasoning, and working memory ^1^. PFC functioning is impaired in cocaine addicts, which is manifest in their inability to make proper decisions when presented with challenges in their environment ^2, 3^. Genetic and environmental factors can influence PFC excitability ^4– 6^, and repeated cocaine alters the excitability of the PFC by biasing neurons towards strong inputs, such as those associated with drug cues, which may diminish cognitive function ^7^. Understanding the mechanism underlying how PFC excitability influences the behavioral responses to psychostimulants is fundamental to learning how to reverse these maladaptive alterations in order to treat addiction.

Canonical transient receptor potential (TRPC) channels are a family of non-selective cation channels that play a crucial role in modulating neuronal excitability due to their involvement in intracellular Ca ^2+^ regulation ^8^. The TRPC5 isoform has been shown to play a role in dendritic growth and arborization through CaMKII-mediated mechanisms throughout the brain ^9, 10^, as well as the expression of fear conditioning in the amygdala ^11^. TRPC5 channels a) are one of the two most prevalent TRPC channels in the adult rodent brain ^12^; b) are densely expressed in deep layer pyramidal neurons of the PFC ^12^; and c) modulate neuronal persistent activity necessary for working memory and attention ^13^.

Since deep-layer pyramidal neurons of the PFC are known to project to limbic structures that subserve reward, such as the ventral tegmental area, nucleus accumbens, amygdala, laterodorsal tegmentum ^14^, and the rostromedial tegmental nucleus ^15^, TRPC5 channels may influence the ability of cortical networks to exert inhibitory control over these structures. Consequently, motivational and drug reward-seeking behaviors may be affected. In the present data sets, we gathered data from mice that lack functional TRPC5 channels in their forebrain CaMKII-expressing pyramidal neurons to measure their cocaine self-administration behavior as an index of cocaine reward.

## Materials and methods

### Subjects

19 adult (25–30 g) male C3H mice were group-housed until surgery. Mice were maintained in a reverse 12 hr light:dark cycle (lights off at 7:00 am) with access to food and water *ad libitum*. Using the cre-lox system, forebrain specific knock-down of *trpc5* was achieved by crossing floxed *trpc5* mice with mice that express Cre recombinase under the control of the αCaMKII promoter, where Cre transgene expression was restricted to excitatory neurons in the forebrain. Breeding pairs of the floxed *trpc5* mice were initially obtained from Dr. Lutz Birnbaumer at the NIEHS and bred at the Institute for Behavioral Genetics. CaMKII-Cre mice were obtained from UTSW Medical Center, Dallas. All procedures were approved by the Institute for Animal Care and Use Committee of the University of Colorado Boulder.

### Surgery

Prior to behavioral experimentation, mice were anaesthetized with a cocktail of 80 mg/mL ketamine and 6 mg/mL xylazine (Sigma Aldrich) and implanted with intravenous catheters as previously described ^16^. Chronically implanted custom catheters consist of Silastic tubing that is affixed to 23-gauge steel tubing bent at a right angle and inserted into a plastic hypodermic needle hub bound to a circular polyurethane backmount and surgical mesh. Catheters were steam autoclaved and rinsed with 70% ethanol prior to surgery. The skin area on the back and above the jugular vein were shaved and prepared with Betadine scrub, 70% ethanol, and 1% Zephiran to prevent infection. Following incisions and exposure of the right external jugular vein, catheter tubing was channeled subcutaneously from the back out the chest above the exposed vein. Catheter tubing was inserted approximately 7 mm into the vein and secured to the vein and surrounding tissue with sterile suture. Following successful insertion and jugular attachment, the incisions were sutured, stapled, and fixed with Vetbond (3M). The neck of the needle hub contains the 23-gauge tubing that remains capped when animals are not connected to the intravenous self-administration apparatus. Catheters were flushed with heparinized pyrogen-free sterile physiological saline *daily* to detect resistance to flow and patency. If animals’ nose-poking behavior deviated by >20% of mean responding, catheter integrity and access to the jugular vein was examined using 10 mg/kg Sodium Brevital. If animals did not exhibit sedation within 3 seconds they were omitted from the study. Requests for the customized mouse catheter system used in this study should be directed to http://neuro-cloud.net/nature-precedings/pomrenze ^16^.

### Self-administration

Seven days following catheter implantation mice (Saline - *trpc5*-KD ( *n* = 8), *trpc5*-WT ( *n* = 8); Cocaine - *trpc5*-KD ( *n* = 9), *trpc5*-WT ( *n* = 10)) were individually housed in self-administration operant chambers that contain two identical nose-poke portals (active and inactive). For acquisition and maintenance of cocaine (unit dose = 0.75 mg/kg/infusion; compounded in pyrogen-free sterile physiological saline; NIDA) self-administration, mice received continuous reinforcement (fixed-ratio 1) of cocaine paired with a 10-second LED illumination and 10-second time-out following a nose-poke into the “active” portal. Inactive portals yielded no consequence. All studies were done without prior operant training. Infusions of 50 uL were delivered over a 4-second time period. For the data set presented mice were exposed to a 3-hr saline self-administration pretest on the first day. Subsequent cocaine daily 3-hr sessions continued until stable responding (> 20 infusions, < 20% variability in number of infusions across three daily sessions, > 70% discriminative responding in “active” portal vs “inactive” portal) was achieved in both groups. All genotypes were blind to the investigators.

### Dose-response

After acquisition of cocaine self-administration and stable maintenance for ≥ 6 days, mice ( *trpc5*-KD ( *n* = 9), *trpc5*-WT ( *n* = 10)) were challenged with a dose-response schedule of varying unit doses (0.05, 0.1, 0.75, and 2.0 mg/kg/infusion) of cocaine (one unit dose per session). The mean number of cocaine infusions at each unit dose was determined during two separate consecutive sessions.

## Results

**Figure 1.**
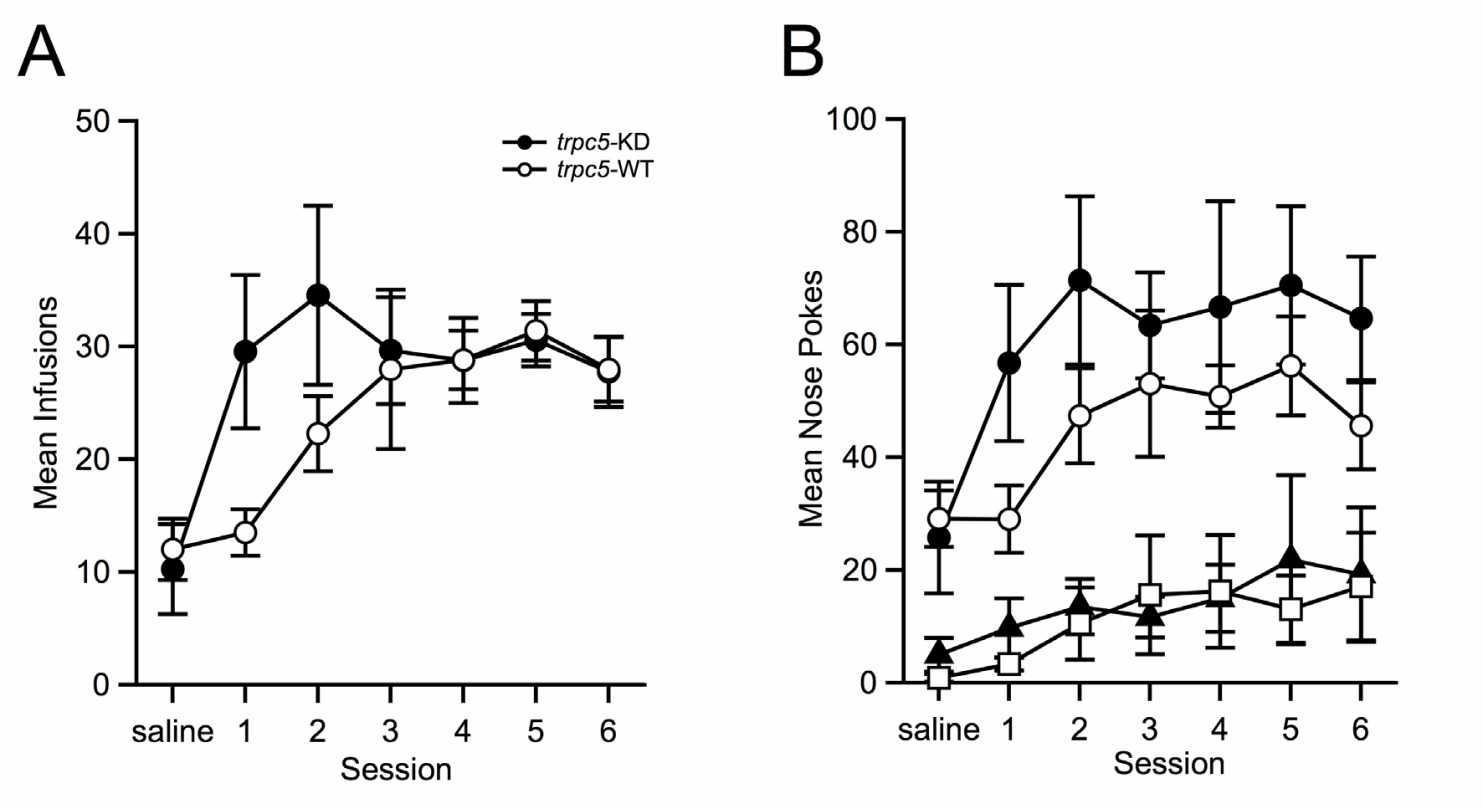
**A**. Mean (± SEM) number of saline (1 session) and cocaine (6 sessions) infusions. Saline responding is similar between genotypes, yet *trpc5*-KD mice exhibit an increased responding on day 1 for cocaine. Saline- *trpc5*-KD ( *n* = 8) versus Saline- *trpc5*-WT ( *n* = 8), Cocaine- *trpc5*-KD ( *n* = 9) versus Cocaine- *trpc5*-WT ( *n* = 10). **B**. Mean (± SEM) number of saline (1 session) and cocaine (6 sessions) nose-poking in active (rewarding) and inactive (non-reinforced) portals. Nose-poking for saline is similar between genotypes, yet *trpc5*-KD poking is significantly elevated on day 1 of responding for cocaine and continues throughout the sessions. Black circles represent *trpc5*-KD active poking, open circles represent *trpc5*-WT active poking, triangles represent *trpc5*-KD inactive poking, squares represent *trpc5*-WT inactive poking.

All mice were exposed to the self-administration chambers to nose-poke for light and saline on their first day of training to establish their baseline levels of exploratory behavior between treatment groups. Cocaine replaced saline thereafter and mice were trained to self-administer cocaine (0.75 mg/kg/infusion) without prior operant training for natural rewards (food pellets). Mice acquired cocaine self-administration by meeting our stated criteria and subsequently exhibited stable self-administration maintenance for ≥ 6 days. WT and *trpc5*-KD mice demonstrated identical cocaine responding during the maintenance phase. The *trpc5*-KD mice reached the criteria by the first session and continued stable responding for the duration of the experiments, whereas WT took several days to catch up to KD responding. WT and *trpc5*-KD mice exhibited similar responding for saline, yet infusions earned by *trpc5*-KD surpassed WT for cocaine on the first session ( Figure 1a). The mouse *trpc5*-KD nose-poking showed a similar pattern on the first session, surpassing WT nose-pokes ( Figure 1b).

**Figure 2.**
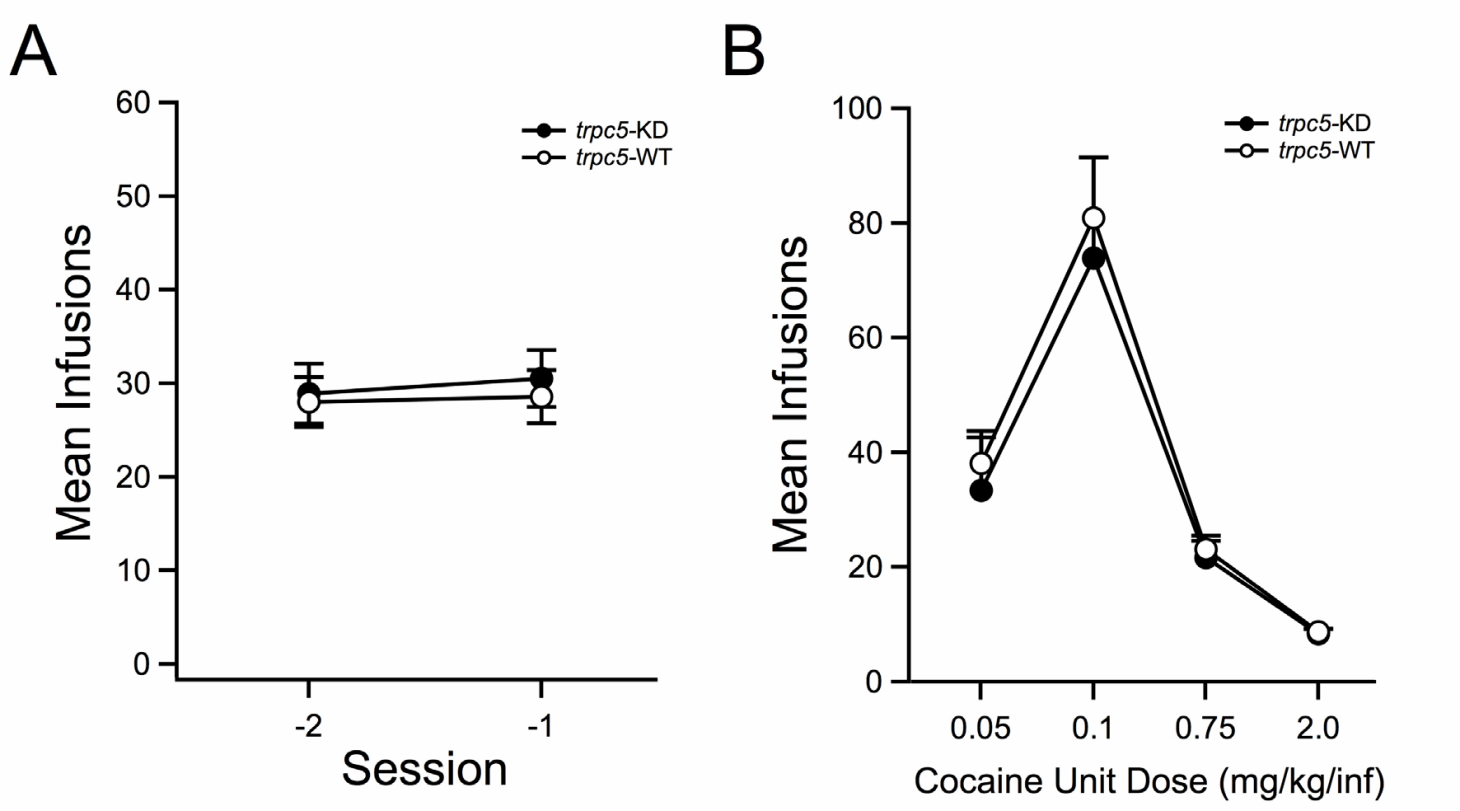
**A**. Mean (± SEM) number of infusions for the last two self-administration sessions prior to the dose-response challenge are similar between *trpc5*-KD and WT mice. Cocaine- *trpc5*-KD ( *n* = 9) versus Cocaine- *trpc5*-WT ( *n* = 10). **B**. Mean (± SEM) number of infusions for 2 sessions at each indicated cocaine dose. Dose-response curves reveal similar responding to varying unit doses of cocaine between genotypes.

After the maintenance phase of self-administration training, separate groups of mice were taken through a dose-response. The sessions leading up to the dose response tests demonstrate similar responding between genotypes ( Figure 2a). Dose-response functions demonstrated no difference between genotypes ( Figure 2b).

Number of infusions and active/inactive nose-pokes for saline or first 6 days of cocaine This data set represents the number of infusions (1a) and the frequency of nose-pokes in the active/inactive portals (1b) for each subject for the saline pretest and for the first 6 days of cocaine responding for mice of each genotype. TRPC5+/+ = wild-type; TRPC5-/- = knock-down; C = Cocaine; Act = Active nose-poke portal; Inact = Inactive nose-poke portal

Cocaine infusions earned before and during the dose-response challenge This data set represents the number of cocaine infusions each subject earned for the last two days preceding (2a), and for the two consecutive sessions during (2b), the dose-response challenge for mice of each genotype. TRPC5 +/+ = wild-type; TRPC5 -/- = knock-down; "-2" = second to last day leading up to dose-response challenge; "-1" = last day leading up to dose-response challenge; S(0.05) = session with dose 0.05 mg/kg/infusion; S(0.1) = session with dose 0.1 mg/kg/infusion; S(0.75) = session with dose 0.75 mg/kg/infusion; S(2.0) = session with does 2.0 mg/kg/infusion; S1 = first daily session with respective dose; S2 = second daily session with respective dose
