# Supplementary material for: Cocaine self-administration in mice with forebrain knock-down of trpc5 ion channels
Source: F1000Res. 2013 Feb 15;2:53. [Version 1] doi: 10.12688/f1000research.2-53.v1 (PMC3790609; doi:10.12688/f1000research.2-53.v1)
Supplement: Peer review history

# Peer review: Referee response for version 1

**Published:** 2013-03-07

**Authors:** Micky Marinelli

**Affiliations:** 1 Rosalind Franklin University of Medicine and Science, North Chicago, IL, USA

This is an interesting study, showing the role of TRPC5 channels in the forebrain, on cocaine self-administration. The experiments are described well. The dose-response curve is convincing, as it shows the characteristic inverted-U-shaped curve. In line with the journal’s guidelines, no statistical results and/or interpretations are provided for any result. However, this makes it difficult to assess the reliability of data, so my comments address this concern.

An analysis of drug intake using repeated measures ANOVA with genotype as between-subject factor and days (cocaine 1-6) as the repeated measure does not yield any significant effects (effect of genotype, main effect of days, or interaction genotype x days). Only a t test on day 1 yields a significant genotype effect. I therefore understand that the authors refer to an “apparent” initial increase in drug intake (abstract). It would be important to replicate this finding in the future, to ascertain that it is a true result, rather than variability in initial intake across mice.

An analysis of active/inactive hole discrimination using ANOVA with genotype as between-subject factor and days (cocaine 1-6) and hole (active/inactive) as the repeated measure yields a trend for an interaction genotype x hole (p=0.068). It therefore appears that the -/- might have better discrimination. This could be reported. The authors could also consider if there is insufficient power for this difference to be significant.

The animals were trained first with saline. Could this have influenced their subsequent intake, and could the initial difference in intake (day 1 of cocaine) be the consequence of differences in the ability to switch across genotypes?

From the data, it appears that not all mice were tested for initial saline intake. Could this have influenced the results?

The authors should describe (methods) how they ran the dose-response curve (descending doses? Random doses? Etc…)

I have read this submission. I believe that I have an appropriate level of expertise to confirm that it is of an acceptable scientific standard.

# Peer review: Referee response for version 1

**Published:** 2013-02-22

**Authors:** Simon Barak Caine

**Affiliations:** 1 Alcohol & Drug Abuse Research Center, McLean Hospital, Belmont, MA, USA

A nicely conducted study. Note three important reservations regarding interpretation and conclusions: 

- Acquisition studies are notorious for variability, and differences between genotypes were observed only on Day 1 of acquisition and throughout the large majority of the study were identical for the two genotypes; 
- A slight hyperactivity especially under reinforced conditions is a fairly common observation with a variety of manipulations, and nose pokes in Figure 1B are suggestive of such a phenomenon (i.e., higher nose pokes during timeouts as well as during reinforcer availability in the active nose poke hole for KD mice than for WT);
- There are no data addressing whether similar results would be obtained with other reinforcers, such as food.

I have read this submission. I believe that I have an appropriate level of expertise to confirm that it is of an acceptable scientific standard.

# Response

**Published:** 2013-02-27

**Authors:** Donald Cooper

**Affiliations:** University of Colorado, Boulder, USA

We thank Dr. Caine for his helpful critique of our Data article. One goal we had for publishing this Data article in F1000Research was to quickly share some of our ongoing behavioral datasets in order to encourage collaboration with others in the field. Our ultimate goal is to  publish these datasets together with more complete analysis and conclusions as well as other work from our lab or new collaborators. Because we intend on publishing these datasets with additional data and more complete analyses and conclusions we kept the analyses and conclusions here to a minimum. 

**Reviewer Concern: Acquisition studies are notorious for variability.** We certainly agree.  To address this issue we took the following approach.  1. The study was conducted with the experimenter blind to the genotype.  2. We reported 9 knock-down and 10 wild-type male age-matched and litter-mate controls that made it through our lengthy testing procedure including dose-response and progressive ratio test (not reported).  We have done further analysis on mice that self-administered on Day 1 of cocaine acquisition but whose catheters failed during the next 3 weeks of testing.  This brought our numbers up to 12 knock-down and 12 wild-type animals.  The Day 1 cocaine means are now trpc5 KD = 27.75 with a standard deviation of 5.3 and wild-type=14.1 with a standard deviation of 2.0.  This gives an effect size of 3.39 and a statistical power estimate at 1.0.  For behavioral studies a statistical power greater than 0.8 is considered good.  So we are confident that there is a difference between groups on Day 1 of cocaine acquisition that is not due to chance alone although ANOVA and posthoc tests are needed and will be presented in a future complete research article. 

**Reviewer Concern:** **The issue of hyperactivity to a reinforcer**Nose poking for light in mice is a behavior that is easy to learn because of the naturally reinforcing property of light activation triggered by a beam break in the nose poke hole.  To address the issue of general hyperactivity:1. We reported nose poke responding for light and found no differences between genotypes on Day 1 of acquisition (Figure 1).  Active nose pokes were higher than inactive nose pokes indicating discrimination in both groups.  2. There were no differences between genotypes in the inactive nose poke port on Day 1 of cocaine acquisition, a general measure of exploratory activity. 3. Other studies in the laboratory have looked at locomotor activation to i.p injections of a locomotor activating dose of cocaine at 10 mg/kg and found no differences between genotypes (data not shown). In addition, a subthreshold dose of 5 mg/kg cocaine or saline also show no differences between genotypes (data not shown).  This suggest no group differences in cocaine-induced locomotor activation in response to an initial exposure to cocaine.4.  The 4 second duration cocaine infusion time and the 10 second time out likely explains the increase in active nose pokes compared to the number of infusions.  However, the ratio of active pokes to infusions was only about 2.  Both groups had similar (1.5 -2 active pokes/infusion) differences at steady-state responding (days 3-6) once acquisition criteria was reached. Future analysis will include a within session analysis of nose pokes to determine if the trpc5 KD mice showed differential nose-poke behavior during the 3 hour session (e.g. more nose pokes/infusion during the first part or last part of the session).  Furthermore, we plan to examine the ratio of active pokes to infusions across the entire dose response to see if the ratio is dose-dependent.  We will include this data set in a revision of the current Data Article.

**Reviewer Concern:** **Would we observe similar results with other reinforcers?**Although we tested light as an alternative reinforcer it is a very good question whether other consumable natural reinforcers like sucrose would be similar to cocaine on Day 1.  These experiments are underway and will be reported with expanded analysis in a future Research Article together with cocaine conditioned place preference, progressive ratio responding and locomotor activity.We welcome any new suggestions from other reviewers or the community at large!
